# LW-AFC Effects on *N*-glycan Profile in Senescence-Accelerated Mouse Prone 8 Strain, a Mouse Model of Alzheimer’s Disease

**DOI:** 10.14336/AD.2016.0522

**Published:** 2017-02-01

**Authors:** Jianhui Wang, Xiaorui Cheng, Ju Zeng, Jiangbei Yuan, Zhongfu Wang, Wenxia Zhou, Yongxiang Zhang

**Affiliations:** ^1^Department of TCM and Neuroimmunopharmacology, Beijing Institute of Pharmacology and Toxicology, Beijing 100850, China; ^2^State Key Laboratory of Toxicology and Medical Countermeasures, Beijing 100850, China; ^3^Educational Ministry Key Laboratory of Resource Biology and Biotechnology in Western China, Life Sciences College, Northwest University, Xi’an 710069, China

**Keywords:** LW-AFC, traditional Chinese medicine, glycome, senescence-accelerated mouse prone 8 strain, Alzheimer’s disease

## Abstract

Glycosylation is one of the most common eukaryotic post-translational modifications, and aberrant glycosylation has been linked to many diseases. However, glycosylation and glycome analysis is a significantly challenging task. Although several lines of evidence have indicated that protein glycosylation is defective in Alzheimer’s disease (AD), only a few studies have focused on AD glycomics. The etiology of AD is unclear and there are no effective disease-modifying treatments for AD. In this study, we found that the object recognition memory, passive avoidance, and spatial learning and memory of senescence-accelerated mouse prone 8 (SAMP8) strain, an AD animal model, were deficient, and LW-AFC, which was prepared from the traditional Chinese medicine prescription Liuwei Dihuang decoction, showed beneficial effects on the deterioration of cognitive capability in SAMP8 mice. Forty-three and 56 *N*-glycan were identified in the cerebral cortex and serum of SAMP8 mice, respectively. The *N*-glycan profile in SAMP8 mice was significantly different from that of senescence accelerated mouse resistant 1 (SAMR1) strains, the control of SAMP8 mice. Treatment with LW-AFC modulated the abundance of 21 and 6 *N*-glycan in the cerebral cortex and serum of SAMP8 mice, respectively. The abundance of (Hex)3(HexNAc)5(Fuc)1(Neu5Ac)1 and (Hex)2(HexNAc)4 decreased in the cerebral cortex and serum of SAMP8 mice compared with SAMR1 mice, decreases that were significantly correlated with learning and memory measures. The administration of LW-AFC could reverse or increase these levels in SAMP8 mice. These results indicated that the effects of LW-AFC on cognitive impairments in SAMP8 mice might be through modulation of *N*-glycan patterns, and LW-AFC may be a potential anti-AD agent.

Alzheimer’s disease (AD) is a neurodegenerative disease with characteristic pathological hallmarks of neuron loss and accumulation of senile plaques and neurofibrillary tangles, resulting in gradual cognitive decline. For AD, disease-modifying treatments remain beyond reach, and the etiology of the disease is uncertain. Glycosylation is one of the most common and complex types of posttranslational modifications, which provides substantial proteome diversity in eukaryotic cells [1, 2]. Mounting evidence shows that these pathological hallmarks of AD are extensively modified by glycosylation, and alterations in glycosylation patterns influence the pathogenesis and progression of AD.

Aberrant alterations in glycosylation on amyloid precursor protein (APP) [3-7], beta-site amyloid precursor protein-cleaving enzyme 1 (BACE1) [8], presenilin (PS), nicastrin, microtubule-associated protein Tau [9-11], cholinesterase (AChE) [12, 13], and transferrin (Tf) [14, 15] have been demonstrated in AD [16]. For example, heparan sulfate proteoglycans (HSPGs) and chondroitin sulfate proteoglycans (CSPGs) have been associated with extracellular β-amyloid (Aβ) senile plaques in the brain [17]. The *N*-glycosylation of acetylcholinesterase (AChE) affected enzymatic activity, as well as its biosynthesis [18]. In additional, significant alteration of cerebrospinal fluid (CSF) *N*-glycome profiling was found in patients with AD or mild cognitive impairment (MCI), consisting of a decrease in the overall sialylation degree and an increase in species bearing bisected GlcNAc [19]. The serum *N*-glycan NA2F level was substantially decreased in patients with AD, but not in controls, and significantly correlated with the CSF Aβ(1-42) and Tau phosphorylated at threonine 181 (P-tau(181P)) levels [20]. The low content of terminal GlcNac and sialic acid in plasma alpha-1-antichymotrypsin (ACT) (a secondary component of amyloid plaques) in patients with AD appeared to be related to progressive cognitive deterioration [21]. There were lower abundances of complex galactosylated and sialylated forms of IgG-Fc glycosylation in the plasma of patients with AD compared to controls and patients with stable mild cognitive impairment (SMCI) or progressive (P)MCI [22]. The expression levels of the serum and CSF protein N-glycans, including bisect-type and multiply branched glycoforms, were increased significantly in patients with AD. The levels of some gangliosides, including GM1, GM2, and GM3, also appeared to be altered in the brain and serum samples of patients with AD when compared with those of normal control groups [23]. These findings have contributed to the discovery and characterization of AD biomarkers and a more detailed understanding of AD biology.

The senescence-accelerated mouse prone 8 (SAMP8) strain is a spontaneous animal model of accelerated aging. The phenotypes of the SAMP8 mouse resemble the symptoms of patients with late-onset and age-related sporadic AD [24]. The classical phenotypic characteristics of SMAP8 mice are progressive cognitive decline and neurodegenerative changes, with a relatively low incidence of other aging phenotypes [24-27]. The cognitive behavioral, pathological, therapeutic, neurochemical, and biochemical features and age-dependent neurodegeneration of SAMP8 mice may substantially mirror the phenotype of sporadic AD with a multifactorial heterogeneous background, and SAMP8 mice are considered a robust model of AD [28-32]. In order to further understand the mechanisms underlying the molecular pathology of AD and develop an effective therapeutic intervention for AD, the *N*-glycan profile in the cerebral cortex and plasma and the learning and memory behavior of SAMP8 mice were investigated, and the specific *N*-glycans correlating with cognitive ability were identified. Furthermore, we found LW-AFC, an herbal medicine, possessed beneficial effects on the deterioration of learning and memory and had a role in modulating *N*-glycan in SAMP8 mice.

## MATERIALS AND METHODS

### Preparation of LW-AFC

LW-AFC was prepared from the Liuwei Dihuang decoction and includes a polysaccharide fraction (LWB-B), a glycoside fraction (LWD-b), and an oligosaccharide fraction (CA-30). The Liuwei Dihuang decoction was prepared as previously described by Yang et al. [33], Zhang et al. [34, 35], and Cheng et al. [36, 37]. Traditional Chinese herbs, including *Radix rehmannia, Fructus corni, Rhizoma dioscoreae, Rhizoma alismatis, Cortex moutan radicis*, and *Poria cocos* were purchased from Beijing Tong-Ren-Tang drug store and subjected to pharmacognostic identification before preparing the decoction. The supernatant filtered from the Liuwei Dihuang decoction was concentrated into a quintessence. The quintessence was then extracted in ethanol to produce the supernatant (LWD), and the sediment left in the deionized water was concentrated into the dried polysaccharide fraction (LWB-B). LWD was concentrated and the ethanol was removed. LWD was then dissolved in deionized water and eluted in turn by deionized water and 30% ethanol on macroporous adsorptive resins. The 30% ethanol elution of LWD was cryodesiccated into a glycoside fraction (LWD-B), and the water elution of LWD was concentrated and eluted in turn by 5% ethanol and 30% ethanol on an active carbon absorption column. The 30% ethanol elution was then concentrated, the ethanol was removed, and the elution was cryodesiccated into the oligosaccharide fraction (CA-30). Finally, LW-AFC was composed of 20.3% polysaccharide fraction (LWB-B), 15.1% glycosides fraction (LWD-B), and 64.6% oligosaccharide fraction (CA-30) by dry weight.

### Animals and drug administration

The original SAMP8 mice and control senescence accelerated mouse resistant 1 (SAMR1) strains were kindly provided by Dr. T. Takeda at Kyoto University, Japan. The mice were maintained in the Beijing Institute of Pharmacology and Toxicology under standard housing conditions (room temperature 22 ± 1°C and humidity of 55 ± 5%) with a 12-h light/12-h dark cycle and free access to water and food. Six-month-old male SAMP8 and SAMR1 mice were randomly separated into 3 groups of 3 mice. LW-treated SAMP8 mice were orally administered 1.6 g/kg/day LW-AFC for 5 months. Untreated SAMP8 mice, as negative controls, and SAMR1 mice, as positive controls, were given equal volumes of distilled water. After administration of LW-AFC for 90 consecutive days, animals underwent novel object recognition tests, Morris-water maze tests, and step-down tests. Following the behavioral experiments, the cerebral cortices were isolated and blood serum was collected from all animals for N-glycan profile analysis. The animal treatment, husbandry, and experimental protocols in this study were approved by the Institutional Animal Care and Use Committee (IACUC) of the National Beijing Center for Drug Safety Evaluation and Research (NBCDSER).

### Novel object recognition test

The procedure of novel object recognition test was according to Bevins & Besheer (2006) [38] and Xu et al. (2015) [39]. The apparatus was placed in an illuminated soundproof room. The procedure included 3 phases: habituation, training, and testing. For 20 minutes per day for 2 consecutive days, the animals were allowed to freely explore the vacant chamber and become familiar with the testing environment. On the third day, two identical objects (sample object A and B) were placed in the box. Each mouse was then allowed to explore the objects for 16 min, then returned to its home cage. The training-to-testing intervals were 1 h and 24 h for testing short-term and long-term object recognition memory, respectively. After the training-to-testing interval, the mouse was placed in a similar chamber and one of the two identical objects was switched for a new one (novel object C). The test session lasted 4 min. The object exploration time (the length of time during which the mouse directed its nose to the object within 2 cm, pawed, or sniffed the object) for objects A, B, and C was recorded and the discrimination index was calculated.

### Morris water maze test

The Morris water maze test was conducted according to Vorhees & Williams (2006) [40] and Hu et al. (2012) [41]. This behavioral task included hidden-platform training (spatial learning) and probe trial (spatial memory) sessions. In the hidden-platform training session, the mouse was allowed 4 daily trials in the presence of the platform, for 5 subsequent days. In these sessions, mice were placed into the pool facing the wall in one of the four quadrants. When the mouse located the platform, it was allowed to stay on the platform for 10 s. If the mouse did not locate the platform within 60 s, it was placed on the platform for 10 s. In the probe trial session, the platform was removed, and the mouse was allowed to swim to search it for 60 sec. The escape latency (the time taken to find the hidden platform) in the hidden-platform training sessions and the escape latency (the first time that the mice crossed the removed platform), time in the target quadrant, and number of crossings of the removed platform in the probe trial sessions were recorded and analyzed.

### Step-down test

Step-down tests were conducted according to He et al. (2010), Luo et al., (2012), and Shi et al. (2010) [42-44]. The step-down test apparatus was composed of a hyaline acrylic chamber with a galvanized grid floor; a rubber platform was placed in the center of the chamber. During the adaptation period, mouse was allowed to freely explore the box, without the platform, to become acquainted with the training environment. In the learning trial (1st day), the mouse was placed on the rubber platform, where it could avoid the electric shock (36 V, AC); the mouse received a shock if it stepped down from the platform (error). The learning course was performed for 10 min. In the testing trials, 2 and 24 h after the learning trial, similar procedures were performed. The testing time was 3 min. Latency was recorded as the time before the mice attempted to step down.

### N-glycan release and purification

The serum samples were dialyzed for 48 h and lyophilized into powder. Cerebral cortex tissue was homogenized in RIPA buffer, then centrifuged to collect the supernatant, which was then dialyzed for 48 h and lyophilized into powder. The powder was denatured in 5% sodium dodecyl sulfate and 0.4 mol/L dithiothreitol at 100°C for 10 min. After cooling to room temperature, 30 µL of phosphate buffer (1.9 g phosphoric acid dissolved in 10 mL deionized water, pH 7.5), 30 µL 10% NP-40, and 1 µL (500 units) of PNGaseF at 37°C were added for 24 h to release N-glycan. N-glycan released by PNGaseF was loaded onto C18 columns activated by 100% methyl cyanide and equilibrated by deionized water; N-glycan samples were then eluted using deionized water. The samples were subsequently loaded onto carbographs activated by 100% methyl cyanide and equilibrated by deionized water. The cartridges were washed with deionized water, and N-glycans were eluted using 2 mL of 25% methyl cyanide containing 0.1% trifluoroacetic acid. Samples were dried in vacuo and reconstituted in deionized water prior to analysis.

### MALDI FT-ICR MS and MS/MS Analysis

Mass spectra were recorded on an FT-ICR MS with an external source ProMALDI (Varian, Palo Alto, CA) equipped with a 7.0 Tesla magnet. The ProMALDI was equipped with a pulsed Nd:YAG laser operating at 355 nm. Internal calibration was done using pre-identified N-glycan peaks, allowing mass accuracy of 10 ppm or better. 2,5-dihydroxy-benzoic acid was used as a matrix (5 mg/100 mL in 50% ACN:H_2_O) for both positive and negative modes. Sodium chloride (NaCl) (0.01 M in 50% ACN:H_2_O) was used as a cation dopant for the positive ion mode. The N-linked glycans were thus seen either as [M+Na]^+^ or [M-H]^-^ in the positive or negative ion modes, respectively. Three spectral acquisitions, each with 10 laser shots, were acquired on the 10%, 20%, and 40% aqueous ACN fractions of a sample, giving a total of nine spectra per time point or per sample. Tandem mass spectrometry using collision-induced dissociation (CID) and infrared multiphoton dissociation (IRMPD) was performed to confirm N-linked glycan compositions.

### Quantification using an Internal Standard

To quantify N-linked glycan in the serum and cerebral cortex of all animals, β-cyclodextrin ([M+Na]^+^, m/z 1157.25; [M-H]^-^, m/z 1133.25) was used as an internal standard. Samples were doped with a fixed amount of β-cyclodextrin and their mass spectral intensities were surveyed for possible neutral (mannose, fucose, and complex-type) and acidic (sialic acid) oligosaccharide masses relative to β-cyclodextrin. The intensities of glycan were then scaled to the intensities of β-cyclodextrin to yield corrected intensities.

### Data Analysis

Mass spectra were acquired and calibrated using IonSpec Omega version 8.0 (Varian). Calibration was done internally, with less than 5 ppm mass error. Oligosaccharides were identified in the first pass using an in-house program called Glycan Finder written in Igor Pro version 5.04B (Wavemetrics), using exact masses with a tolerance of 10 ppm. A biological filter was then applied following Cooper’s method [45], as explained in our previous work [46, 47]. One-way analysis of variance of the glycan intensities was then performed to compare between time points and groups. P values less than 0.05 were considered significant. The Dixon’s Q-test was used to determine outliers.

### Statistical analysis

All data were expressed as mean ± S.D. GraphPad Prism 6.0 (GraphPad Software, Inc., La Jolla, CA, USA) was used to plot and analyze data. Data between two groups were compared by Student’s t-test. Two-tailed Pearson correlation analyses were employed to measure the correlation between the relative abundance of N-glycan and mouse cognitive behavioral data. P < 0.05 was taken as statistically significant.

## RESULTS

### Treatment with LW-AFC ameliorated the cognitive deterioration of SAMP8 mice

**Figure 1.**
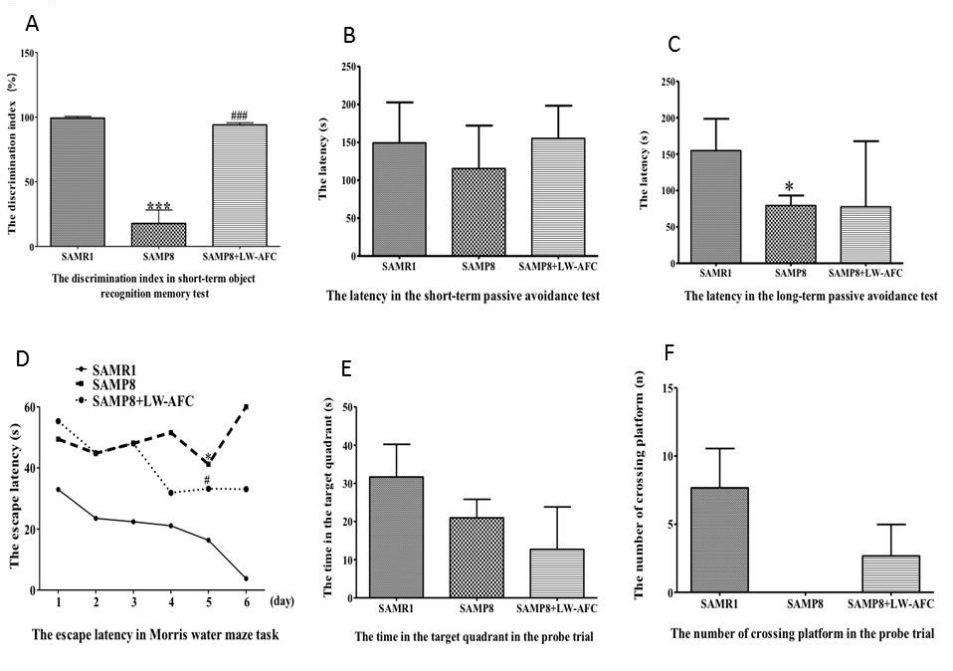
**The treatment of LW-AFC ameliorated cognitive deterioration of SAMP8 mice.** The discrimination index **(A)** in the object recognition memory test. The latency in the short-term **(B)** and long term **(C)** passive avoidance test respectively in step-down test. The escape latency **(D)** in Morris water maze test. The time in the target quadrant **(E)** and the number of crossing platform **(F)** in the probe trial of Morris water maze test. *p < 0.05, ***p < 0.001, comparing with SAMR1 mice. ^#^*p* < 0.05, ^###^*p* < 0.001, comparing with SAMP8 mice. Mean or mean ± S.D., n=3, Student’s *t*-test.

The discrimination index (the percentage of time spent with the novel object with respect to the total exploration time) in the novel object recognition test indicated that the deficits in short-term object recognition memory in SAMP8 mice were significantly reversed by treatment with LW-AFC (Fig. 1A), but the long-term object recognition memory of SAMP8 mice was not changed (Fig. 1B). The results of step-down tests showed that, compared with SAMR1 mice, the long-term passive avoidance of SAMP8 mice was deficient; LW-AFC treatment had no effect (Fig. 1D). Short-term passive avoidance did not differ among SAMR1 mice, SAMP8 mice, and LW-AFC-treated SAMP8 mice (Fig. 1C). The Morris water maze test was employed to measure the ability of spatial learning and memory of mice. In the learning task, untreated SAMP8 mice showed longer escape latencies than SAMR1 mice on the final day, but treatment with LW-AFC decreased this latency (Fig. 1E). In the probe trial, SAMP8 mice did not cross the platform, but LW-AFC administration increased the number of platform crossings (Fig. 1H). The escape latency (Fig. 1F) and time in the target quadrant (Fig. 1G) in the probe trial did not differ among three groups. These data indicated that treatment with LW-AFC ameliorated cognitive deterioration in SAMP8 mice.

### The effect of LW-AFC on N-glycan profile in the cerebral cortex of SAMP8 mice

**Figure 2.**
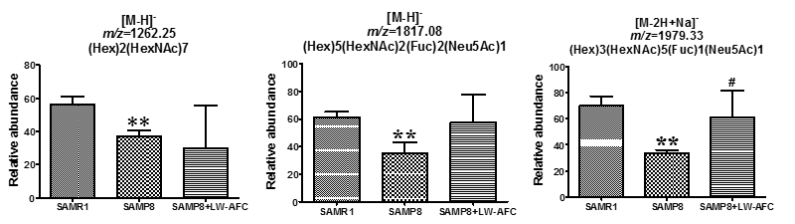
**The effect of LW-AFC on *N*-glycan in the cerebral cortex of SAMP8 mice.** **p<0.01, comparing with SAMR1 mice. ^#^*p*<0.05, comparing with SAMP8 mice. Mean ± S.D., n=3, Student’s *t*-test.

In the cerebral cortex of SAMP8 mice, 43 *N*-glycans were identified (Table 1). Among the 43 *N*-glycans, 9 only existed in the cerebral cortex of SAMR1 mice: No. 1, 2, 7, 10, 19, 21, 29, 25, and 42. Five *N*-glycans were specifically present in the cerebral cortex of SAMP8 mice, including No. 4, 13, 16, 30, and 34 (Table 1). Compared to SAMR1 mice, the abundances of (Hex)2(HexNAc)7, (Hex)5(HexNAc)2(Fuc)2(Neu5Ac)1 and (Hex)3 (HexNAc)5(Fuc)1(Neu5Ac)1 decreased in the cerebral cortex of SAMP8 mice (Table 1) (Fig. 2). Fifteen *N*-glycans were not found in the cerebral cortices of SAMP8 mice, but treatment with LW-AFC could induce SAMP8 mice to produce them: No. 1, 2, 3, 5, 7, 9, 10, 12, 14, 19, 21, 22, 29, 31, and 33. Conversely, administration of LW-AFC inhibited production of *N*-glycans, including No. 8, 13, 16, 24, and 30, in SAMP8 mice (Table 1). Treatment with LW-AFC also significantly increased the abundance of (Hex)3(HexNAc)5(Fuc)1(Neu5Ac)1 in the cerebral cortex of SAMP8 mice (Table 1) (Fig. 2). These data indicated that the *N*-glycan profile in the cerebral cortex of SAMP8 mice was different from that of SAMR1 mice and the treatment of LW-AFC could modulate it.

**Table 1:** The effect of LW-AFC on *N*-glycan profile in the cerebral cortex of SAMP8 mice

No.	Observed mass(*m/z*)	Type	Proposed composition	Relative abundance in SAMR1group	Relative abundance in SAMP8 group	Relative abundance in SAMP8+LW-AFC group
1	1013.25	[M+Na]^+^	(Hex)6	7.65±13.24	undetectable	16.26±14.09
2	1079.33	[M+Na]^+^	(Hex)3(HexNAc)2(Fuc)1	10.88±18.84	undetectable	10.95±18.97
3	1095.33	[M+Na]^+^	(Hex)4(HexNAc)2	undetectable	undetectable	4.86±8.42
4	1136.42	[M+Na]^+^	(Hex)3(HexNAc)3	undetectable	12.99±22.49	10.11±17.52
5	1175.33	[M+Na]^+^	(Hex)7	undetectable	undetectable	7.30±12.65
6	1257.42	[M+Na]^+^	(Hex)5(HexNAc)2	127.72±56.63	74.41±17.89	146.88±47.49
7	1273.25	[M+K]^+^	(Hex)5(HexNAc)2	25.72±22.29	undetectable	29.15±13.14
8	1298.42	[M+Na]^+^	(Hex)4(HexNAc)3	11.09±19.20	11.36±19.68	undetectable
9	1337.33	[M+Na]^+^	(Hex)8	undetectable	undetectable	14.68±14.15
10	1339.33	[M+Na]^+^	(Hex)3(HexNAc)4	10.14±17.56	undetectable	4.29±7.44
11	1419.33	[M+Na]^+^	(Hex)6(HexNAc)2	50.61±18.67	14.22±24.64	54.64±20.40
12	1435.42	[M+K]^+^	(Hex)6(HexNAc)2	undetectable	undetectable	10.38±17.99
13	1444.50	[M+Na]^+^	(Hex)4(HexNAc)3(Fuc)1	undetectable	13.88±24.04	undetectable
14	1460.17	[M+Na]^+^	(Hex)5(HexNAc)3	undetectable	undetectable	4.15±7.18
15	1485.42	[M+Na]^+^	(Hex)3(HexNAc)4(Fuc)1	32.70±5.87	12.04±20.86	6.77±11.73
16	1501.42	[M+Na]^+^	(Hex)4(HexNAc)4	undetectable	11.04±19.12	undetectable
17	1581.42	[M+Na]^+^	(Hex)7(HexNAc)2	28.42±7.76	9.34±16.18	28.58±9.66
18	1647.50	[M+Na]^+^	(Hex)4(HexNAc)4(Fuc)1	17.51±15.50	9.83±17.03	7.33±12.69
19	1663.33	[M+Na]^+^	(Hex)5(HexNAc)4	8.06±13.97	undetectable	7.89±13.67
20	1688.50	[M+Na]^+^	(Hex)3(HexNAc)5(Fuc)1	30.02±6.34	23.39±20.31	16.62±16.84
21	1743.58	[M+Na]^+^	(Hex)8(HexNAc)2	21.40±20.30	undetectable	22.37±19.43
22	1809.50	[M+Na]+	(Hex)5(HexNAc)4(Fuc)1	undetectable	undetectable	3.56±6.16
23	1905.58	[M+Na]^+^	(Hex)8(HexNAc)2	18.10±15.90	8.17±14.15	21.80±7.98
24	1931.25	[M+H]+	(Hex)5(HexNAc)4(Neu5Ac)1	7.13±12.35	7.13±12.35	undetectable
25	951.25	[M-H]^-^	(Hex)2(HexNAc)3	24.89±21.74	undetectable	undetectable
26	1031.25	[M-H]^-^	(Hex)5(HexNAc)1	12.25±21.23	74.61±98.09	14.80±25.64
27	1072.25	[M-H]^-^	(Hex)4(HexNAc)2	94.92±19.20	39.98±35.23	106.49±33.32
28	1126.17	[M-2H]^2-^	(Hex)5(HexNAc)4(Neu5Gc)2	43.35±5.17	31.62±27.55	66.33±34.43
29	1154.33	[M-H]^-^	(Hex)2(HexNAc)4	32.17±28.32	undetectable	25.84±44.75
30	1193.25	[M-H]^-^	(Hex)6(HexNAc)1	undetectable	46.49±80.53	undetectable
31	1234.33	[M-H]^-^	(Hex)5(HexNAc)2	undetectable	undetectable	38.34±35.22
32	1331.25	[M-H]^-^	(Hex)2(HexNAc)2(Fuc)2(Neu5Ac)1	99.76±15.50	49.15±49.66	100.26±54.13
33	1396.33	[M-H]^-^	(Hex)6(HexNAc)2	undetectable	undetectable	51.89±7.59
34	1420.17	[M-H]^-^	(Hex)4(HexNAc)3(Fuc)1	undetectable	12.29±21.28	17.07±29.57
35	1475.17	[M-H]^-^	(Hex)2(HexNAc)2(Fuc)1(Neu5Ac)2	52.37±5.96	14.01±24.27	33.73±30.02
36	1493.25	[M-H]^-^	(Hex)3(HexNAc)2(Fuc)2(Neu5Ac)1	19.85±34.37	44.43±6.95	72.83±27.07
37	1559.25	[M-H]^-^	(Hex)2(HexNAc)6	62.39±4.18	32.95±30.02	29.41±50.93
38	1655.08	[M-H]^-^	(Hex)4(HexNAc)2(Fuc)2(Neu5Ac)1	61.86±6.32	24.18±21.17	40.48±39.45
39	1721.25	[M-H]^-^	(Hex)3(HexNAc)6	26.91±23.32	19.03±18.33	18.57±32.17
40	1762.25	[M-H]^-^	(Hex)2(HexNAc)7	56.02±4.75	36.83±3.67**	29.76±26.05
41	1817.08	[M-H]^-^	(Hex)5(HexNAc)2(Fuc)2(Neu5Ac)1	61.43±3.91	35.11±7.83**	57.42±20.29
42	1905.50	[M-H]^-^	(Hex)6(HexNAc)3(Neu5Gc)1	11.22±19.43	undetectable	undetectable
43	1979.33	[M-2H+Na]^-^	(Hex)3(HexNAc)5(Fuc)1(Neu5Ac)1	69.68±7.52	33.75±2.30**	60.87±20.82^#^

Compositions and observed masses of the *N*-glycan were identified and integrated by MALDI FT-ICR MS and MS/MS Analysis. Relative abundance was scaled to the intensities of an internal standard β-cyclodextrin to yield a corrected intensity. Hex=Hexoses, HexNAc=*N*-acetyl hexosamine, NeuAc=*N*-acetylneuraminic acid, NeuGc=*N*-glycolylneuraminic acid, Fuc=fucose.

** *p*<0.01, comparing with SAMR1 mice.

# *p*<0.05, comparing with SAMP8 mice. Data is mean ± S.D., n=3, Student’s *t*-test.

**Figure 3.**
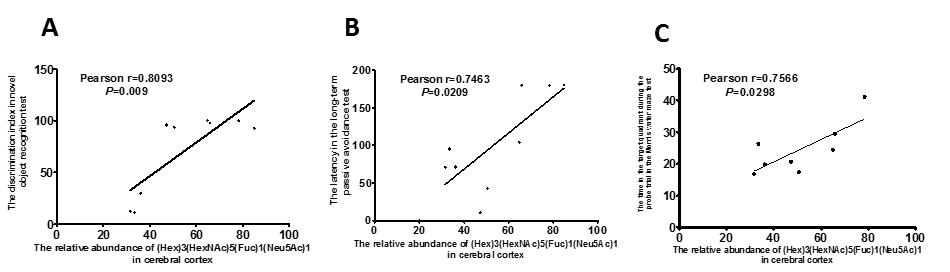
**Correlation between relative abundance of (Hex)3(HexNAc)5(Fuc)1(Neu5Ac)1 in cerebral cortex and ability of learning and memory of SAM mice.** n=8-9, two-tailed Pearson analysis, confidence interval 95%.

In order to investigate whether the ability of learning and memory was related to *N*-glycan, we further calculated the correlation between the abundance of *N*-glycan and the cognitive abilities of all mice using Pearson correlation analyses. Results showed that the abundance of (Hex)3(HexNAc)5(Fuc)1(Neu5Ac)1 in the cerebral cortex of SAMP8 mice was significantly correlated with the discrimination index in the short-term object recognition memory test (Fig. 3A), latency in the long-term passive avoidance test (Fig. 3B), and time in the target quadrant in the probe trial of the Morris water maze test (Fig. 3C). These data indicated that the abundance of (Hex)3(HexNAc)5(Fuc)1(Neu5Ac)1 in the cerebral cortex of SAMP8 mice was significantly correlated with cognitive capability and LW-AFC could affect it (Fig. 2 and 3).

**Table 2:** The effect of LW-AFC on *N*-glycan profile in the serum of SAMP8 mice

No	Observed mass (*m/z*)	Type	Proposed composition	Relative abundance in SAMR1group	Relative abundance in SAMP8 group	Relative abundance in SAMP8+LW-AFC group
1	933.33	[M+Na]^+^	(Hex)3(HexNAc)2	undetectable	7.04±12.19	undetectable
2	996.75	[M+2Na]^2+^	(Hex)5(HexNAc)4(Neu5Gc)1	undetectable	28.90±26.32	37.75±4.03
3	1095.33	[M+Na]^+^	(Hex)4(HexNAc)2	6.30±10.90	15.68±5.48	6.86±11.87
4	1136.42	[M+Na]^+^	(Hex)3(HexNAc)3	undetectable	23.96±3.30	26.46±3.28
5	1150.67	[M+2Na]^2+^	(Hex)5(HexNAc)4(Neu5Gc)2	17.44±17.09	16.10±14.21	29.05±6.92
6	1153.17	[M+2Na]^2+^	(Hex)4(HexNAc)5(Neu5Ac)2	61.19±17.03	26.26±23.47	40.84±1.76
7	1176.17	[M+2Na]^2+^	(Hex)3(HexNAc)6(Fuc)2(NeuAc)1	38.48±34.46	undetectable	undetectable
8	1257.42	[M+Na]^+^	(Hex)5(HexNAc)2	46.49±8.79	46.27±8.77	56.65±5.40
9	1298.42	[M+Na]^+^	(Hex)4(HexNAc)3	49.74±15.62	45.20±7.87	50.10±4.63
10	1339.33	[M+Na]^+^	(Hex)3(HexNAc)4	undetectable	5.28±9.15	undetectable
11	1419.33	[M+Na]^+^	(Hex)6(HexNAc)2	31.77±27.61	45.25±6.72	56.32±3.73
12	1460.17	[M+Na]^+^	(Hex)5(HexNAc)3	45.45±12.42	19.22±18.88	34.72±0.70
13	1485.42	[M+Na]^+^	(Hex)3(HexNAc)4(Fuc)1	81.39±20.23	38.55±34.08	64.20±7.39
14	1501.42	[M+Na]^+^	(Hex)4(HexNAc)4	undetectable	17.91±15.90	undetectable
15	1518.17	[M+H]^+^	(Hex)8(HexNAc)1	55.06±16.90	26.38±10.08	21.88±18.97
16	1565.08	[M+Na]^+^	(Hex)6(HexNAc)2(Fuc)1	6.02±10.42	3.72±6.44	3.69±6.40
17	1581.42	[M+Na]^+^	(Hex)7(HexNAc)2	undetectable	13.04±2.49	10.44±9.04
18	1605.25	[M+Na]^+^	(Hex)4(HexNAc)3(Neu5Gc)1	24.90±8.64	18.99±5.91	22.95±2.36
19	1622.42	[M+Na]^+^	(Hex)6(HexNAc)3	undetectable	4.86±8.41	undetectable
20	1647.5	[M+Na]^+^	(Hex)4(HexNAc)4(Fuc)1	96.53±22.57	64.58±20.94	83.08±5.96
21	1663.33	[M+Na]^+^	(Hex)5(HexNAc)4	59.67±52.99	83.38±15.36	90.29±7.08
22	1679.33	[M+K]^+^	(Hex)5(HexNAc)4	8.47±7.69	10.21±8.86	15.71±1.67
23	1727.17	[M+Na]^+^	(Hex)7(HexNAc)2(Fuc)1	15.11±13.22	7.45±6.73	11.26±1.17
24	1743.58	[M+Na]^+^	(Hex)8(HexNAc)2	4.47±7.75	11.86±1.94	14.03±0.28
25	1767.25	[M+Na]^+^	(Hex)5(HexNAc)3(Neu5Gc)1	25.39±8.54	15.53±5.70	18.03±0.88
26	1783.25	[M+K]^+^	(Hex)5(HexNAc)3(Neu5Gc)1	5.60±9.69	undetectable	undetectable
27	1809.5	[M+Na]^+^	(Hex)5(HexNAc)4(Fuc)1	39.45±11.40	26.14±6.94	20.95±18.16
28	1825.25	[M+Na]^+^	(Hex)5(HexNAc)4(Fuc)1	39.23±11.05	20.37±7.72*	24.47±1.78
29	1885.33	[M+H]^+^	(Hex)4(HexNAc)6	22.17±6.13	10.78±4.56	13.77±0.50
30	1905.58	[M+Na]^+^	(Hex)8(HexNAc)2	8.91±8.47	7.33±6.39	4.80±8.32
31	1970.33	[M+Na]^+^	(Hex)5(HexNAc)4(Neu5Gc)1	67.45±27.07	62.19±20.73	70.39±11.18
32	1986.33	[M+K]^+^	(Hex)5(HexNAc)4(Neu5Gc)1	152.52±56.67	72.77±39.77	90.35±1.85
33	951.25	[M-H]^-^	(Hex)2(HexNAc)3	4.96±0.93	1.23±2.12	3.54±1.15
34	1075.67	[M-4H+2Na]^2-^	(Hex)3(HexNAc)6(Neu5Gc)1S1	8.43±9.42	10.04±2.54	6.31±5.47
35	1126.17	[M-2H]2^-^	(Hex)5(HexNAc)4(Neu5Gc)2	41.86±19.00	26.77±9.13	32.75±7.60
36	1154.33	[M-H]^-^	(Hex)2(HexNAc)4	7.43±1.09	4.75±0.85*	6.99±0.30^#^
37	1199.67	[M-2H]^2-^	(Hex)5(HexNAc)4(Fuc)1(Neu5Gc)2	7.04±2.27	2.80±2.42	3.27±2.83
38	1234.33	[M-H]^-^	(Hex)5(HexNAc)2	undetectable	1.22±2.11	3.21±2.78
39	1258.08	[M-H]^-^	(Hex)3(HexNAc)3(Fuc)1	14.94±5.64	7.02±1.15*	9.37±4.52
40	1309.17	[M-2H]^2-^	(Hex)6(HexNAc)5(Neu5Gc)2	40.73±23.56	undetectable	25.63±16.28
41	1316.25	[M-H]^-^	(Hex)3(HexNAc)4	10.93±2.93	5.63±4.89	5.74±4.97
42	1401.17	[M-2H]^2-^	(Hex)5(HexNAc)4(Neu5Ac)4	15.02±5.23	6.11±5.34	7.88±7.23
43	1420.17	[M-H]^-^	(Hex)4(HexNAc)3(Fuc)1	undetectable	3.68±6.37	12.46±2.49
44	1462.67	[M-2H]^2-^	(Hex)6(HexNAc)5(Neu5Gc)3	13.58±6.66	4.83±4.23	4.95±4.29
45	1497.25	[M-2H+Na]^-^	(Hex)2(HexNAc)2(Fuc)1(Neu5Ac)2	2.93±5.07	undetectable	undetectable
46	1559.25	[M-H]^-^	(Hex)2(HexNAc)6	7.98±7.02	2.91±5.03	undetectable
47	1581.33	[M-H]^-^	(Hex)4(HexNAc)3(Neu5Gc)1	17.66±8.88	18.33±2.48	20.61±0.87
48	1659.42	[M-2H+Na]^-^	(Hex)3(HexNAc)2(Fuc)1(Neu5Ac)2	3.08±5.34	undetectable	undetectable
49	1721.25	[M-H]^-^	(Hex)3(HexNAc)6	12.27±2.89	5.12±4.82	9.86±1.00
50	1743.5	[M-H]^-^	(Hex)5(HexNAc)3(Neu5Gc)1	18.29±4.14	14.35±2.89	15.31±0.23
51	1784.33	[M-H]^-^	(Hex)4(HexNAc)4(Neu5Gc)1	22.81±21.09	25.56±5.60	26.36±0.82
52	1823.08	[M-2H+Na]^-^	(Hex)3(HexNAc)6S1	12.27±4.66	13.93±0.74	8.54±7.40
53	1845.17	[M-3H+2Na]^-^	(Hex)3(HexNAc)6S1	15.59±14.82	15.11±2.77	17.33±4.83
54	1905.5	[M-H]^-^	(Hex)6(HexNAc)3(Neu5Gc)1	18.15±3.62	11.89±2.23*	14.37±0.77
55	1946.5	[M-H]^-^	(Hex)5(HexNAc)4(Neu5Gc)1	109.10±42.94	95.91±23.40	112.52±8.99
56	1968	[M-2H+Na]^-^	(Hex)5(HexNAc)4(Neu5Gc)1	2.78±4.82	6.09±0.46	4.34±3.76

Compositions and observed masses of the *N*-glycan were identified and integrated by MALDI FT-ICR MS and MS/MS Analysis. Relative abundance were scaled to the intensities of an internal standard β-cyclodextrin to yield a corrected intensity. Hex=Hexoses, HexNAc=*N*-acetyl hexosamine, NeuAc=*N*-acetylneuraminic acid, NeuGc=*N*-glycolylneuraminic acid, Fuc=fucose.

** *P*<0.01, comparing with SAMR1 mice.

# *P*<0.05, comparing with SAMP8 mice. Data is mean ± S.D., n=3, Student’s *t*-test.

### The effect of LW-AFC on N-glycan profile in the serum of SAMP8 mice

**Figure 4.**
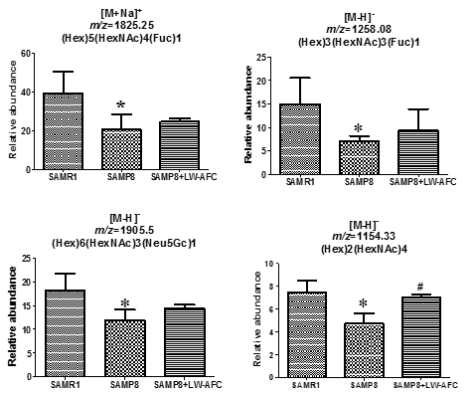

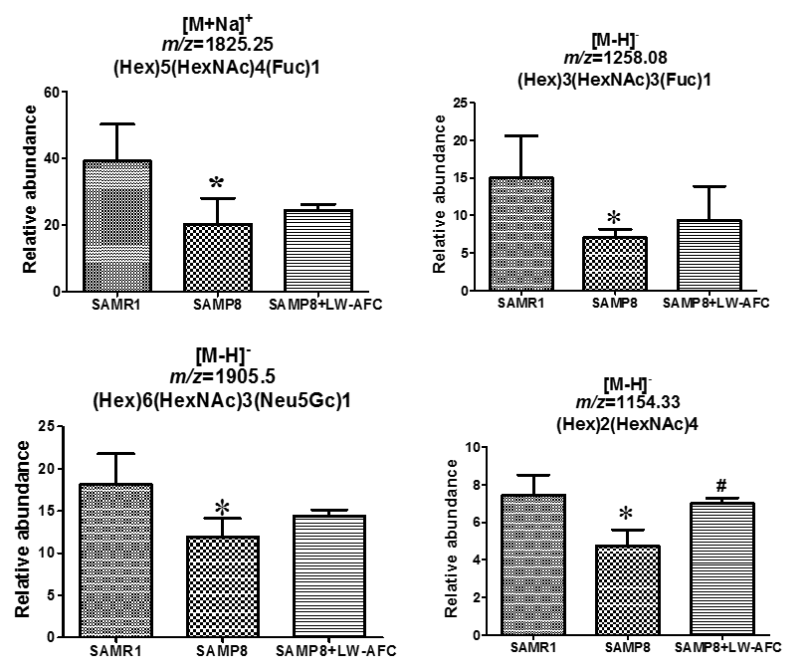
**The effect of LW-AFC on *N*-glycan in the serum of SAMP8 mice.** *p<0.05, comparing with SAMR1 mice. ^#^*p*<0.05, comparing with SAMP8 mice. Mean ± S.D., n=3, Student’s *t*-test.

Fifty-six *N*-glycans were identified in the serum of SAMP8 mice (Table 2). Among these 56 *N*-glycans, only 5 were found in the serum of SAMR1 mice: No. 7, 26, 40, 45, and 48. Nine *N*-glycans were specifically found in the serum of SAMP8 mice: No. 1, 2, 4, 10, 14, 17, 19, 38, and 43 (Table 2). Compared to SAMR1 mice, the abundances of (Hex)5(HexNAc)4(Fuc)1, (Hex)3(HexNAc)3(Fuc)1, (Hex)6(HexNAc)3(Neu5Gc)1 and (Hex)2(HexNAc)4 were lower in the serum of SAMP8 mice (Table 2) (Fig. 4). The *N*-glycan (Hex)6(HexNAc)5(Neu5Gc)2 was not found in the serum of untreated SAMP8 mice, but treatment with LW-AFC could induce SAMP8 mice to produce it. LW-AFC treatment also inhibited production of (Hex)3(HexNAc)2, (Hex)3(HexNAc)4, (Hex)4(HexNAc)4, and (Hex)6(HexNAc)3 in SAMP8 mice (Table 2). Treatment with LW-AFC significantly increased the abundance of (Hex)2(HexNAc)4 in the serum of SAMP8 mice (Table 2) (Fig. 4). The *N*-glycan pattern in the serum of SAMP8 mice differed from that of SAMR1 mice, and treatment with LW-AFC regulated it.

**Figure 5.**
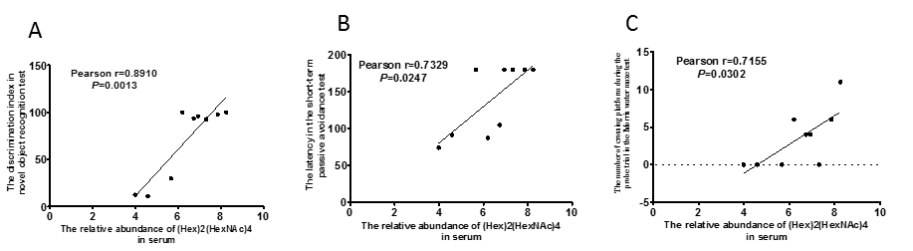
**Correlation between relative abundance of (Hex)2(HexNAc)4 in serum and ability of learning and memory of SAM mice.** n=9, two-tailed Pearson analysis, confidence interval 95%.

Furthermore, we calculated the correlation between the abundance of *N*-glycan in serum and the learning and memory abilities of all mice using Pearson correlation analyses. The abundance of (Hex)2(HexNAc)4 in the serum of SAMP8 mice was significantly correlated with the discrimination index in the short-term object recognition memory test (Fig. 5A), latency in the short-term passive avoidance test (Fig. 5B), and number of platform crossings in the probe trial of the Morris water maze test (Fig. 5C). The abundance of (Hex)2(HexNAc)4 in the serum of SAMP8 mice was significantly correlated with learning and memory ability and LW-AFC could affect it in SAMP8 mice (Fig. 4 and 5).

## DISCUSSION

Glycosylation plays critical roles in numerous biological processes and some disease etiologies [48-54]. Alterations in glycosylation may be good biomarkers for monitoring health conditions [55-57]. Several lines of evidence have indicated that protein glycosylation is defective in AD [3-18, 22], including APP [3-7], BACE1 [8], Tau [9-11], AChE [12, 13], and Tf [14, 15]. However, only a few AD glycomics studies have been reported [19-23, 58]. More comprehensive and detailed studies may therefore lead to valuable insights into AD etiology and provide novel therapeutic interventions for AD.

SAMP8 mice are considered a robust model for exploring the etiopathogenesis of sporadic AD and a plausible experimental model for developing therapeutic approaches for AD [32]. The present study identified the specific *N*-glycosylation patterns of SAMP8 mice and found beneficial effects of LW-AFC on cognitive deterioration and aberrant alterations of *N*-glycan in SAMP8 mice. We also found that the abundance of (Hex)3(HexNAc)5(Fuc)1(Neu5Ac)1 in the cerebral cortex and (Hex)2(HexNAc)4 in the serum of SAMP8 mice correlated with cognition. A similar study was reported by Gizaw et al. [59], who found that total glycome expression levels were significantly different between Huntington’s disease (HD) transgenic and control group mice, and the brain glycome and expression levels were significantly gender specific when compared to those of other tissues and serum [59]. Additionally, the structural profiles of the major N-glycans released from glycoproteins and the total expression levels of the glycans were mostly similar between the brain tissues of patients with AD patients and normal controls. We also found that the N-glycosylation patterns in the cerebral cortex were mostly similar between SAMP8 and SAMR1 mice.

It is well known that human congenital disorders of ganglioside biosynthesis and congenital disorders of glycosylation (CDGs) result in intellectual disability [60]. The predominant form of sialic acid in human milk is *N*-acetylneuraminic acid (Neu5Ac), and sialic acid is an essential nutrient for brain development and cognition [61]. Sialylation regulates brain structure and function [62]. Until now, no study indicated a correlation between (Hex)3(HexNAc)5(Fuc)1(Neu5Ac)1 or (Hex)2(HexNAc)4 and learning and memory. Additionally, the mechanisms of action of some drugs may modulate glycosylation patterns; for example, olanzapine treatment in patients with schizophrenia resulted in changes in the glycosylation machinery associated with the biosynthesis of abundant serum proteins [63]. This study indicated that improvement in cognitive impairments as a result of LW-AFC treatment might occur through regulation of *N*-glycosylation patterns in SAMP8 mice.

This study achieved comprehensive profiling of *N*-glycosylation patterns in SAMP8 mice and found a correlation between (Hex)3(HexNAc)5(Fuc)1(Neu5Ac)1 or (Hex)2(HexNAc)4 and cognition. Moreover, that treatment with LW-AFC could improve cognitive impairments and modulate *N*-glycan pattern suggested that LW-AFC might be a potential anti-AD agent.
